# Mitochondrial Transcription Termination Factor 27 Is Required for Salt Tolerance in *Arabidopsis thaliana*

**DOI:** 10.3390/ijms22031466

**Published:** 2021-02-02

**Authors:** Deyuan Jiang, Jian Chen, Zhihong Zhang, Xin Hou

**Affiliations:** State Key Laboratory of Hybrid Rice, College of Life Sciences, Wuhan University, Wuhan 430070, China; dyjiang@whu.edu.cn (D.J.); 2015202040070@whu.edu.cn (J.C.); zzh@whu.edu.cn (Z.Z.)

**Keywords:** *Arabidopsis*, mTERF27, MORF8, salt stress, mitochondrial morphology

## Abstract

In plants, mTERF proteins are primarily found in mitochondria and chloroplasts. Studies have identified several mTERF proteins that affect plant development, respond to abiotic stresses, and regulate organellar gene expression, but the functions and underlying mechanisms of plant mTERF proteins remain largely unknown. Here, we investigated the function of *Arabidopsis* mTERF27 using molecular genetic, cytological, and biochemical approaches. *Arabidopsis* mTERF27 had four mTERF motifs and was evolutionarily conserved from moss to higher plants. The phenotype of the mTERF27-knockout mutant *mterf27* did not differ obviously from that of the wild-type under normal growth conditions but was hypersensitive to salt stress. mTERF27 was localized to the mitochondria, and the transcript levels of some mitochondrion-encoded genes were reduced in the *mterf27* mutant. Importantly, loss of mTERF27 function led to developmental defects in the mitochondria under salt stress. Furthermore, mTERF27 formed homomers and directly interacted with multiple organellar RNA editing factor 8 (MORF8). Thus, our results indicated that mTERF27 is likely crucial for mitochondrial development under salt stress, and that this protein may be a member of the protein interaction network regulating mitochondrial gene expression.

## 1. Introduction

Mitochondria, which originated through the endosymbiosis of an α-proteobacterial ancestor, are considered the “power house” of the cell, providing the necessary energy for cellular function. Mitochondria have their own intrinsic genomes, RNA, and ribosomes. The regulation of mitochondrial genome expression is vital to the coordination of energy demands during particular growth and developmental stages in plants [1]. Plant mitochondria have unique and complex RNA metabolism mechanisms, combining the characteristics of their prokaryotic ancestors with the new features of evolution in eukaryotic hosts [2]. Compared to animals, the mitochondrial genomes of plants are relatively larger [3]. Post-transcriptional mechanisms, including RNA editing, RNA splicing, maturation of transcriptional ends, RNA degradation, and other processing steps play a controlling role in gene expression pattern of mitochondria [4,5,6,7]. During the integration of the endosymbiont into the present-day mitochondrial genome, most mitochondrial genetic content was transferred to the nucleus of the host cell [8]. While plant mitochondria are larger than animal genomes, they retain only a minor portion of their ancestral genomes. In *Arabidopsis thaliana*, the mitochondrial genome consists of 57 mitochondrial genes encoding for subunits of the respiratory chain, and the cytochrome maturation complexes, 40 ribosomal proteins, tRNAs, and rRNAs have been reported [2,9,10]. Thus, thousands of originally mitochondrial genes are now expressed under central control of the nucleus, and their protein products are subsequently imported into the mitochondria. As a result, mitochondrial biogenesis relies on the coordinated expression of organellar and nuclear genomes [2,9,11].

The mitochondrial transcription termination factor (mTERF) protein family is a key player affecting gene expression in plastid and mitochondrial genomes [12]. mTERF proteins are characterized by a modular architecture consisting of tandem repeats of a conserved 30-amino acid sequence, known as the mTERF motif [12]. In animals, the mTERF family has only four members, mTERF1–4, which are all localized to the mitochondria [13]. By contrast, more than 30 different mTERF proteins are widely distributed across plant nuclear genomes [14].

Thirty-five mTERF proteins have been identified in *Arabidopsis,* and mutations in several of these proteins have previously been associated with defects in development or stress responses [14,15]. For example, deficiencies in mTERF1/SOLDAT10, mTERF4/BSM/RUG2, mTERF5/MDA1, mTERF6, mTERF9/TWIRT1, mTERF15, or mTERF18/SHOT1 block development [16,17,18,19,20,21], while deficiencies in mTERF5/MDA1, mTERF6, mTERF9/TWIRT1, mTERF10, mTERF11, and mTERF18/SHOT1 affect responses to various abiotic stresses [18,22,23,24]. Most mTERF proteins in plants target the mitochondria and chloroplasts, playing an active part in organellar gene expression and RNA transcription [14,25]. For example, mTERF15 participates in mitochondrial intron splicing, mTERF22 affects the expression of many mitochondrial genes [20,26], and mTERF18/SHOT1 influences the steady-state abundance of various mitochondrial transcripts [18]. In *Chlamydomonas reindhardtii,* the mTERF-like protein MOC1 promotes the termination of anti-sense mitochondrial transcription [27]. However, even though plants have more mTERFs than mammals, the functional network of mTERFs in plants remains little known.

In addition to mTERFs, many other nuclear-encoded protein families, such as pentatricopeptide repeat (PPR) proteins and multiple organellar RNA editing factor (MORF) proteins, also play vital roles in the transcriptional and post-transcriptional regulation of organellar gene expression [9]. Like mTERFs, PPRs, which are characterized by tandem repeats of a degenerate 35-amino acid motif, are a large group of eukaryote-specific nucleic acid binding proteins encoded by the nucleus; PPRs function as RNA-binding proteins and regulate the processing of chloroplastic and mitochondrial RNA [28]. PPRs have also recently been shown to participate in the tolerance of certain stressors, such as salt and other abiotic factors [29]. The *Arabidopsis* genome encodes hundreds of PPR proteins [28].

The *Arabidopsis* genome encodes 10 MORF proteins (also termed RNA-editing factor interacting proteins, RIPs) [30,31]: MORF2, MORF9, and MORF10 are located in plastids; MORF1, MORF3, MORF4, MORF6, and MORF7 are located in mitochondria; and MORF5 and MORF8 are found in both organelles [32]. MORF deficiencies were shown to affect plant development and RNA editing at multiple sites in both mitochondria and plastids, many of which are associated with different individual PPR proteins [30,31,32]. MORFs have also been shown to interact with various PPR proteins, and they can form both homo- and heteromers [30,31,32]. Some MORFs may participate in the response to salt and other abiotic stresses, as well as the development of mitochondria and chloroplasts [33].

In this study, we identify and functionally characterize mTERF27 in *Arabidopsis thaliana*. Cell fluorescence imaging analyses showed that mTERF27 was localized to the mitochondria. Loss of mTERF27 decreased salt tolerance. Quantitative real-time PCR (qRT-PCR) and transmission electron microscope (TEM) analyses showed that defects in mTERF27 compromised mitochondrial gene expression and development under salt stress. Finally, we explored the direct interaction between mTERF27 and MORF8, and showed that mTERF27 interacted with itself to form homomers.

## 2. Results

### 2.1. mTERF27 Is a Mitochondria-Localized mTERF Protein

**Figure 1:**
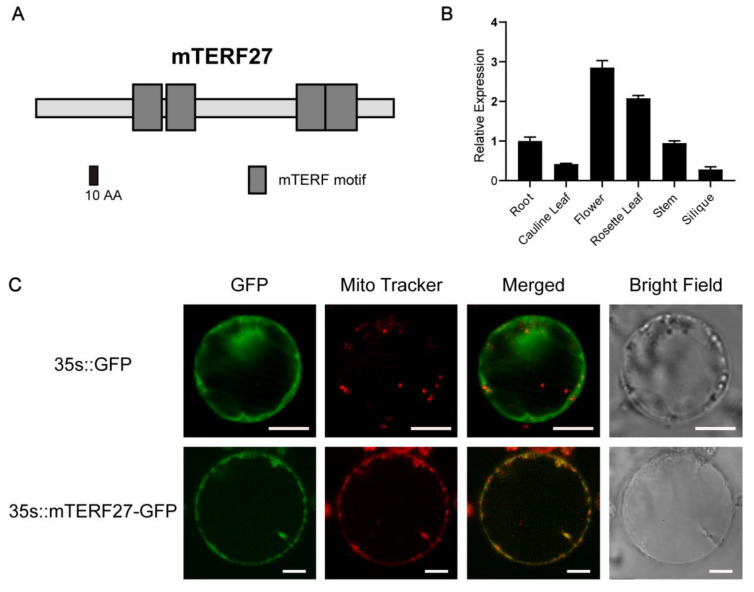
Modular architecture, expression pattern, and subcellular localization of mTERF27. (**A**) Schematic representation of the mTERF27 protein, drawn using SMART. mTERF motifs are shown as gray boxes. (**B**) Tissue-specific expression patterns of mTERF27, determined using qRT-PCR. Data are shown as means ± SD, n = 3. *ACTIN2* was used as an internal control. (**C**) Transient expression of the vectors *35s::GFP* and *35s::mTERF27-GFP* in *Arabidopsis* protoplasts. Mitochondrial locations are shown using MitoTracker Red (Invitrogen). Scale bars = 10 μm.

Land plant genomes have considerably larger numbers of mTERF proteins than other eukaryotes; *Arabidopsis* encodes at least 35 mTERF proteins [12,15]. Nonetheless, few *Arabidopsis* mTERF genes have been characterized in detail. *AT1G21150* (*mTERF27* [15]) is one of the previously unreported mTERF genes. Domain architecture analysis using SMART (http://smart.embl-heidelberg.de) indicated that mTERF27 carried four mTERF motifs (Figure 1A). The homologous features of the *mTERF27* gene sequence were identified using Dicots PLAZA 4.0 (https://bioinformatics.psb.ugent.be/plaza/versions/plaza_v4_dicots/). From genomes of 55 species contained in Dicots PLAZA 4.0 database, 52 BHI (Best-Hits-and-Inparalogs) orthologs of mTERF27 were found (Figure S1A). The phylogenetic tree, constructed based on the 52 orthologs of *mTERF27,* indicated that *mTERF27* were relatively well-conserved across the plant kingdom, from *Physcomitrella patens* to higher plants (Figure S1B).

To investigate the gene expression patterns of *mTERF27* in *Arabidopsis*, we assessed the expression of *mTERF27* in various plant organs using qRT-PCR. The *mTERF27* gene was constitutively expressed in all tissues and organs examined, and the expression level was relatively higher in rosette leaves and flower (Figure 1B).

Most *Arabidopsis* mTERF proteins are found in mitochondria and/or chloroplasts; the mitochondrial localization of the mTERF27 had been preliminarily detected in the guard cells of *Arabidopsis*. [17]. To verify this result, we constructed a vector containing the full length CDS of *mTERF27* fused to GFP. Then, this vector (*35s*::*mTERF27*-*GFP*) and the empty control vector (*35s*::*GFP*) were transiently expressed in separate *Arabidopsis* protoplasts. Florescent localization analysis indicated that mTERF27-GFP signals overlapped well with the MitoTracker signal corresponding to mitochondria (Figure 1C). These results indicated that *mTERF27* encodes a mitochondria-localized mTERF protein.

### 2.2. Disruption of mTERF27 Reduced Arabidopsis Resistance to Salt Stress

**Figure 2:**
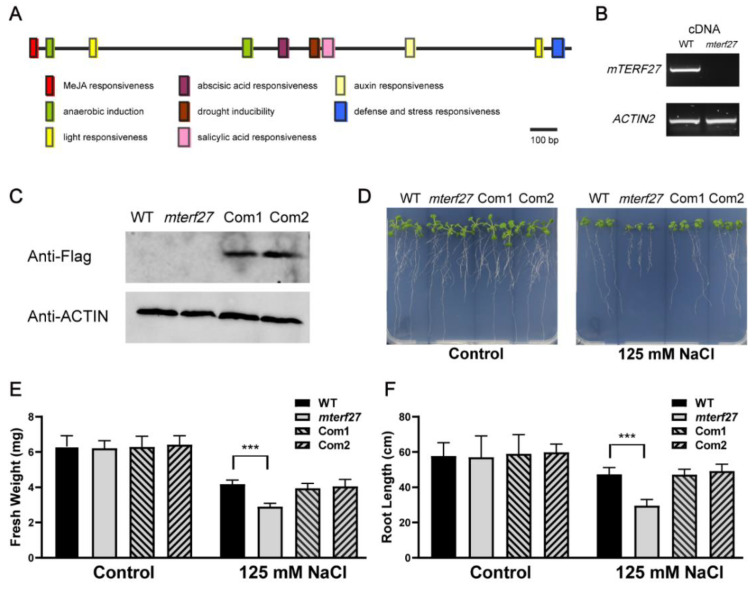
Identification of the *Arabidopsis mterf27* mutant and phenotypes of WT and *mterf27* seedlings under salt stress treatment. (**A**) Cis-acting regulatory elements of mTERF27 analyzed by PlantCARE. The bar represents 100 bp of nucleic acids. (**B**) PCR amplifications showing *mTERF27* expression in WT and *mterf27* plants. *ACTIN2* was used as an internal control. cDNA, complementary DNA. (**C**) Western blots verifying mTERF27-Flag expression in *mTERF27*-complemented lines (Com1 and Com2) based on total proteins extracted using the anti-Flag antibody. Anti-β-actin was used as internal control. (**D**) Fourteen-day-old WT, *mterf27,* Com1, and Com2 plants grown for 10 days in either 1/2 MS medium (Control) or 1/2 MS medium supplemented with 125 mM NaCl. (**E**) Fresh weights and (**F**) root lengths of the WT, *mterf27*, Com1, and Com2 plants shown in Figure 2D. Data shown are means ± SD of three independent experiments. Asterisks show significant differences compared to the WT: ***, *p* < 0.001 (Student’s *t* test).

To explore the cis-acting regulatory elements of *mTERF27*, we used PlantCARE (http://bioinformatics.psb.ugent.be/webtools/plantcare/html/) to analyze the promoter region 2000 bp upstream of the *mTERF27* start codon. As shown in Figure 2A, the existence of cis-acting regulatory elements such as the abscisic acid responsive element, MeJA responsive element, auxin responsive element, salicylic acid responsive element, light responsive element, and a drought-responsive element implied that *mTERF27* might be involved in abiotic stress response. Furthermore, recent studies have suggested that plant mTERFs may play a role in the response to various abiotic stresses, such as salt [33].

To investigate the phenotype of the loss-of-function mutant of mTERF27, we obtained one transfer DNA (T-DNA) insertion line from the Arabidopsis Biological Resource Center (https://abrc.osu.edu): SAIL_902, which putatively carries a T-DNA insertion in the *AT1G21150* (*mTERF27*) gene. PCR and sequencing analyses confirmed the T-DNA insertion site of the SAIL_902 mutant (here referred to as the *mterf27* mutant) (Figure S2A,B). The 1009 bp T-DNA was inserted in the exon of *AT1G21150* (position 7407135 in chromosome 1) in the mutant line (Figure S2C). RT-PCR analyses showed that *mTERF27* transcripts were absent in the homozygous mutant lines but present in the wild-type lines (Figure 2B).

The homozygous mutant plants displayed a wild-type (WT)-like phenotype under control growth conditions (Figure 2D, left), and loss of *mTERF27* did not affect photosynthetic activity (Figure S3). To generate lines complementing the *mTERF27* mutant, the coding region of *AT1G21150* was fused with the Flag tag, and the resulting construct (*35s:*:*mTERF27*-*Flag*) was introduced into homozygous *mterf27* plants. Two independent T1 transgenic plants were identified as complemented lines (referred to as Com1 and Com2). Western-blot analyses indicated that the mTERF27-Flag protein was expressed in both complement lines (Figure 2C).

To clarify the function of mTERF27 in the response of *Arabidopsis* to salt stress, WT, *mterf27*, Com1, and Com2 plants were cultivated on 1/2 MS medium with or without 125 mM NaCl supplementation. Interestingly, when grown in salt-stressed conditions, the *mterf27* mutant displayed reduced growth compared to the WT (Figure 2D, right). To see the details, chlorophyll fluorescence which represents photosynthetic efficiency was examined. The results showed that loss of *mTERF27* did not affect photosynthetic activity even under salt-stressed conditions (Figure S3). In addition, the fresh weight and root length of WT, *mterf27*, Com1, and Com2 seedlings with or without salt stress were measured. While the fresh weights of WT, *mterf27*, Com1, and Com2 seedlings grown under control conditions did not differ significantly, the fresh weight of the *mterf27* seedlings was significantly lower than that of all other seedlings (Figure 2E). Similarly, the root lengths of WT, *mterf27*, Com1, and Com2 seedlings grown under control conditions did not differ significantly, but the root length of the *mterf27* seedlings was significantly lower than the root lengths of all other seedlings (Figure 2F). In these comparisons, Com1 and Com2 seedlings generally displayed phenotypes similar to that of the WT. Thus, we hypothesized mTERF27 participated in salt stress tolerance in *Arabidopsis*.

### 2.3. Disruption of mTERF27 Affected Mitochondrial Gene Transcription and Altered Mitochondrial Morphology

**Figure 3:**
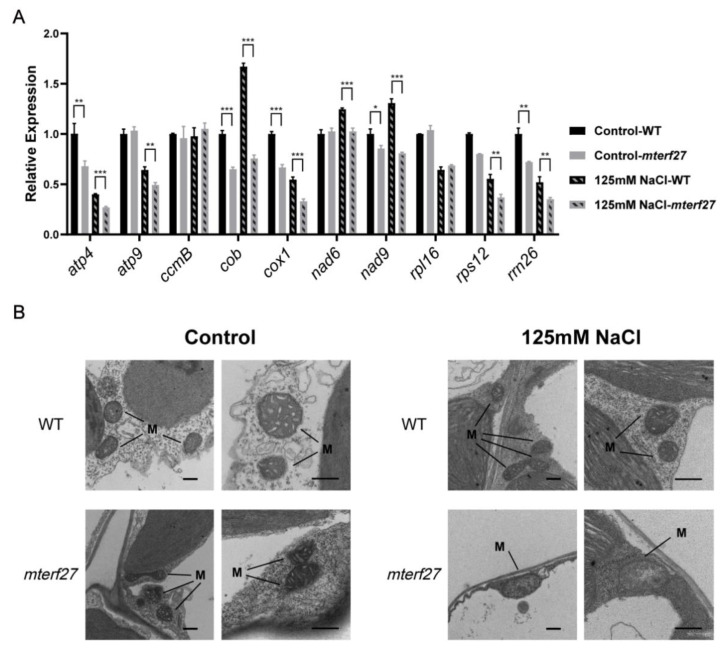
Mitochondrial gene expression levels and mitochondrial morphology in WT and *mterf27* plants. (**A**) Relative expression of mitochondrial genes in 14-day-old WT and *mterf27* plants were grown for 10 days in either 1/2 MS medium (Control) or 1/2 MS medium supplemented with 125 mM NaCl. Relative expression was measured using qRT-PCR, with *ACTIN2* as the internal control. Data represent means ± SD of three independent experiments. Asterisks show significant differences compared to the WT: ***, *p* < 0.001; **, *p* < 0.01; *, *p* < 0.05 (Student’s *t* test). (**B**) TEM images of leaves from plants shown in (**A**). M, mitochondria. Scale bars = 0.5 μm.

mTERF27 is a mitochondria-localized mTERF family protein, which may affect mitochondrial gene transcription. To figure out if salt stress affected the accumulation of certain mitochondrial transcripts in *mterf27* plants, total RNA was extracted from WT and *mterf27* plants grown in control and salt-stressed conditions. RT-qPCR results showed that without salt stress, the transcript levels of *atp4* (subunit of complex V), *cob* (subunit of complex III), *cox1* (subunit of complex IV), *nad9* (subunit of complex I) and *rps12* (Ribosomal protein S12) in the *mterf27* mutant were lower than those in the WT plants, indicating that loss of mTERF27 led to a certain deficiency in the expression of some mitochondrial genes. (Figure 3A). After salt stress, the expression of *atp4*, *atp9* (subunit of complex V), *cox1*, *rps12* and *rrn26* was reduced in the WT plants, while these reductions were pronounced in the *mterf27* mutant plants (Figure 3A). These suggested that mTERF27 affected the expression of some mitochondrial genes in *Arabidopsis*, especially under salt stress. 

Recent reports have indicated that deficiencies in many mTERF proteins influence the development of chloroplasts or mitochondria [29,33]. To evaluate the effects of *mTERF27* mutation on mitochondrial biogenesis, we examined the morphologies of mitochondria from the leaves of WT and *mterf27* plants grown in control or salt-stressed conditions using TEM. Under control growth conditions, TEM images showed that both WT and *mterf27* plants had normally structured mitochondrial cristae with small inner spaces (Figure 3B). In contrast, when WT and *mterf27* plants were grown under salt stress, the *mterf27* mitochondria lacked cristae and had large internal space, while the WT mitochondria showed regular cristae (Figure 3B). This suggested that mTERF27 is required for mitochondrial development under salt stress.

### 2.4. mTERF27 Forms Homomers and Interacts with MORF8

**Figure 4:**
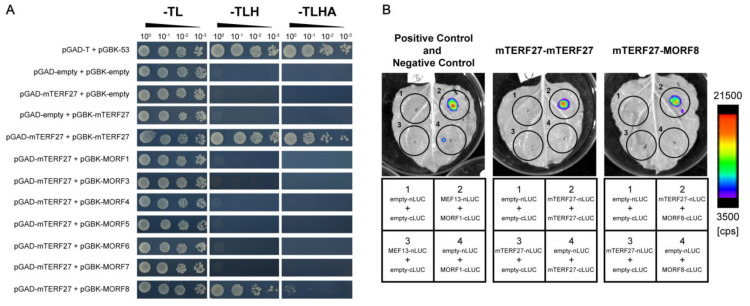
mTERF27 directly interacts with mTERF27 and MORF8. (**A**) Yeast two-hybrid assays showing interactions between mTERF27 and MORF8. pGAD, pGADT7 with the GAL4 activation domain; pGBK, pGBKT7 with the GAL4 DNA binding domain; -TL, SD/-Trp-Leu dropout medium; -TLH, SD/-Trp-Leu-His dropout medium; -TLHA, SD/-Trp-Leu-His-Ade dropout medium. The 53-T interaction were used as positive controls. (**B**) Firefly luciferase complementation assays showing mTERF27-mTERF27 and mTERF27-MORF8 interactions in planta. MEF13-MORF1 interaction were used as positive controls [34]. Color scale represents the luminescent signal intensity measured by cps (counts per second).

Similar to mTERFs, PPRs are a large group of eukaryotic-specific nucleic acid binding proteins encoded by the nucleus, and *Arabidopsis* genome encodes hundreds of PPR proteins. Many PPRs and MORFs are known to interact [30,31,32], so we tested whether mTERF27 interacted with any MORFs in *Arabidopsis.* To investigate this, yeast two-hybrid assays were used to detect interactions between mTERF27 and mitochondria-localized MORFs (i.e., MORF1, MORF3, MORF4, MORF5, MORF6, MORF7 and MORF8). We also used yeast two-hybrid assays to determine whether mTERF27 formed homomers. Growth analyses in selective media (SD-T/-L/-H and SD-T/-L/-H/-A) showed that mTERF27 interacted with MORF8, but not with any other MORFs; mTERF27 also interacted with itself (Figure 4A and Figure S4). These interactions were validated in planta using firefly luciferase complementation imaging assays (Figure 4B). Yeast growth on selective medium and bioluminescence signals produced by the catalysis of luciferin partly reflected that mTERF27 has weaker interaction with MORF8, comparing with its homomer interaction.

## 3. Discussion

Our results showed that the *mterf27* mutants were hypersensitive to salt stress. Under salt stress, the loss of mitochondria-localized mTERF27 disrupted mitochondrial development and caused defects in mitochondrial gene expression. Furthermore, mTERF27 directly interacted with MORF8 and possibly formed homomers.

The mTERF protein family shares several features with the PPR and MORF protein families. For example, PPR and mTERF proteins both harbor tandem repeats of a conserved domain [28], and mutations in both types of proteins have been linked to developmental defects or inhibited stress responses [33]. In addition, PPRs participate in organellar RNA metabolism and function as specific RNA-binding proteins [9]. Similarly, it has been reported that some mTERF proteins bind mitochondrial and chloroplastic nucleic acids in *Arabidopsis* [20,35,36,37]. 

MORFs may provide an ordered spatial connection between PPRs and other proteins [9,32]. Indeed, recent studies have indicated that the MORF8 protein, which is located in both the mitochondria and the chloroplasts [32], directly interacts with multiple PPRs in *Arabidopsis* [30,34,38]. MORF8 is also involved in the establishment and/or mediation of a direct or indirect connection between MEF13 (a PPR protein) and MORF3 [34]. Here, our results suggested that mTERF27 might interact with MORF8 to participate in mitochondrial gene expression and RNA metabolism (Figure 4A,B). Curiously, the interaction between mTERF27 and MORF8 is weaker than mTERF27 homomer interaction (Figure 4A,B). Similar to the model of MEF13-MORF1-MOEF3 interaction [34], other factors may affect the interaction between mTERF27 and MORF8 in mitochondria. However, there is no evidence to show the relation between the mTERF homomer interaction and mTERF-MORF interaction so far. The molecular mechanism remains unclear. 

In plants, mutations in some mTERF genes lead to paleness, significant retardations in growth and development, and even arrested embryogenesis [17,20,21]. However, mutant lines defective in some other mTERFs exhibit less severe growth and developmental defects or display hypersensitivity to abiotic stress [22,26,37]. Previous studies showed that deficiency of mTERF5/MDA1, mTERF6, mTERF9/TWIRT1, mTERF10, and/or mTERF11 resulted in altered response to salt stress and/or ABA treatment in the mutants [19,22,23,39]. A recent study reported that mTERF9 and mTERF5 are negative regulators of salt tolerance, and have contributions to plastid gene expression and retrograde signaling in *Arabidopsis thaliana* [24]. Our results showed that similar to mTERF9 and mTERF5, mTERF27 is also involved in plant salt response and mitochondrial gene expression.

Although the expression level of some mitochondrial genes was lower in *mterf27* mutants compared with WT, there was no visible defects of *mterf27* mutant under normal growth conditions. Previous studies have indicated that mTERF7, mTERF22, and mTERF27 have close phylogenetic relationships [26]. Therefore, under normal growth conditions, mTERF7 and mTERF22 might complement the loss function of mTERF27 partly. However, mTERF27 may play a critical role in plant salt response. Under salt stress conditions, *mterf27* mutant displayed retarded growth phenotype. mTERFs may play diverse roles in organelles. Various pairs of transcription factors, including mTERFs, may have redundant or complementary function networks in plant mitochondria and chloroplasts. Indeed, some mTERF proteins in plants are involved in the transcription termination of chloroplast genes [35,37]. However, recent reports showed that plant mTERFs may have a more complicated mechanism in organelle gene expression [36,40,41,42].

The characterization of mTERF27 in this study helps to clarify plant organellar gene expression in response to salt and other abiotic stress. Our work also provides a basis for further analyses of the mTERF−MORF protein−protein interaction network and investigations of its functional relevance.

## 4. Materials and Methods

### 4.1. Plant Materials and Growth Conditions

*Arabidopsis thaliana*, ecotype Columbia-0 (Col-0), was grown in a growth chamber under 16 h of light at 22 °C and 8 h of darkness at 20 °C. To grow seedlings on agar plates, surface-sterilized seeds were planted on 1/2 MS medium containing 1.0% (*w*/*v*) sucrose and 0.8% (*w*/*v*) agar, cold-treated for 2 days, and transferred to a growth chamber. To test seedling salt tolerance, seeds were planted on normal 1/2 MS medium, cold treated for 2 days, grown for 2 days under normal conditions, transferred to either unmodified 1/2 MS medium (control growth conditions) or 1/2 MS medium supplemented with 125 mM NaCl (salt-stressed growth conditions), and then grown for 10 days in the growth chamber. To grow seedlings in soil, sown seeds were cold-treated for 2 days and then transferred to a green room under same growth conditions (16 h of light at 22 °C and 8 h of darkness at 20 °C). *Nicotiana benthamiana* was cultured in autoclaved vermiculite in a green room under a 16 h light/8 h dark photoperiod at 25 °C; 4–5-week-old plants were used for transient expression analysis.

### 4.2. Plant Transformation

The coding region of the *mTERF27* gene was amplified from total RNA using reverse-transcriptase PCR (RT-PCR). The resulting cDNA was cloned into vector pCAMBIA1300 to produce a construct expressing the Flag-tagged mTERF27 protein. *Agrobacterium tumefaciens* strain GV3101 was used for transformation. The constructs were transferred into *mterf27* mutants using the floral dip method, and transgenic plants were identified using hygromycin resistance analysis, PCR genotyping, and western blots.

### 4.3. Chlorophyll Fluorescence Measurements

Chlorophyll fluorescence imaging and analysis were performed using a chlorophyll imaging system (FluorCam FC 800-C/1010, PSI), with photosynthetic parameters determined as described previously [43]. Before each measurement, plants were dark-adapted for 20 min. The F_v_/F_m_ ratio was defined as (F_m_ − F_o_)/Fm. The nonphotochemical quenching (NPQ) was calculated as (F_m_ − F′m)/F′_m_, where F_m_ is the maximum fluorescence value in the dark-adapted state; F′_m_ is the maximum fluorescence value in any light-adapted state; and F_o_ is the minimal fluorescence value in the dark-adapted state.

### 4.4. RNA Isolation and Quantitative Reverse-Transcriptase PCR (RT-qPCR)

Total RNA was isolated with the leaves of WT, *mterf27*, Com1, and Com2 plants using a RNeasy Plant Mini Kit (Qiagen). cDNA was synthesized using a PrimeScript RT reagent Kit with gDNA Eraser (Takara). qPCRs were performed with the 7300Plus real-time PCR system (ABI) using TB Green *Premix Ex Taq* II (Tli RNaseH Plus) (Takara). The *ACTIN2* gene was used as an endogenous control. Primers used to detect the mitochondrial transcripts were designed as previously described [26].

### 4.5. Protein Preparation and Western Blots

Total protein samples were prepared and western blots were performed following a previous study [43]. For immunoblotting analysis, we separated equal amounts of protein sample on 10% SDS PAGE gels and transferred them to nitrocellulose membranes. After blocking nonspecific binding with 5% milk, we subsequently incubated the blot with specific primary antibodies generated against the indicated proteins and secondary horseradish peroxidase conjugated antibodies (Abbkine). Signals were detected using the SuperSignal™ West Pico PLUS Chemiluminescent Substrate (Thermo Scientific) according to the manufacturer’s protocol. The primary antibodies used were Anti-Flag (Sigma-Aldrich, #F3165) and Anti-β-actin (Abbkine, #A01050-2).

### 4.6. Microscopy

To transiently express *mTERF27* in *Arabidopsis* protoplasts, the full-length CDS of *mTERF27* was cloned into pHBT-sGFP plasmids as previously described [44]. Mesophyll protoplasts were extracted from 4-week-old darkness-treated *Arabidopsis* leaves and transformed with the GFP plasmids as previously described [44]. MitoTracker Red (Invitrogen) was used to specifically dye the mitochondria. The organelle and GFP signals were detected with a confocal microscope (TCS SP8, Leica). The excitation and emission wavelengths were as follows: GFP, excitation at 488 nm and emission at 510–540 nm; MitoTracker, excitation at 644 nm and emission at 650–680 nm.

For TEM analysis, leaves from WT and *mterf27* seedlings were prepared as described previously [45]. Leaves were observed and imaged using an HT7800 Compact-Digital TEM system (Hitachi).

### 4.7. Protein Interaction Assays

Yeast two-hybrid assays were performed using the yeast strain Y2H Gold (Clontech), following the manufacturer’s instructions. For construction of the Gateway entry clones, PCR products were inserted into pDONR207 via BP reactions (Gateway BP clonase enzyme mix; Invitrogen), then cloned into the expression vectors (pGBKT7 or pGADT7) which contain the attR1-CmR-ccdB-attR2 fragment via LR reactions (Gateway LR clonase enzyme mix; Invitrogen) as previously described [46]. These vectors were transformed into Y2H Gold yeast (Clontech). The transformants were grown on SD/-Trp-Leu, SD/-Trp-Leu-His, and SD/-Trp-Leu-His-Ade dropout selective culture-media.

To perform firefly luciferase complementation imaging assays, the coding regions of the target genes were fused with either nLUC or cLUC and cloned into the pCAMBIA1300 vector as previously described [47]. These vectors were transformed into *A. tumefaciens.* The positive clones were injected into *N. benthamiana* as previously described [47], the bioluminescent signals were detected by NightSHADE LB985 system (Berthold).

## Data Availability

Data are available on request to the corresponding author.

## Supplementary Materials

The following are available online at https://www.mdpi.com/1422-0067/22/3/1466/s1, Figure S1. Amino acid sequences alignment and Neighbor-joining phylogenetic tree of mTERF27 ortholog genes in plants; Figure S2. Sequences flanking the transfer DNA (T-DNA) insertion point in the *mterf27* mutant; Figure S3. Chlorophyll fluorescence analysis of the wild-type (WT) and *mterf27* plants shown in Figure 2D; Figure S4. Negative controls for the yeast two-hybrid assay used to determine protein interactions; Table S1. Primers used in this study.
