# Supplementary material for: Mitochondrial Transcription Termination Factor 27 Is Required for Salt Tolerance in Arabidopsis thaliana
Source: Int J Mol Sci. 2021 Feb 2;22(3):1466. doi: 10.3390/ijms22031466 (PMC7867191; doi:10.3390/ijms22031466)
Supplement: Supplementary file 1 [file ijms-22-01466-s001.pdf]

A

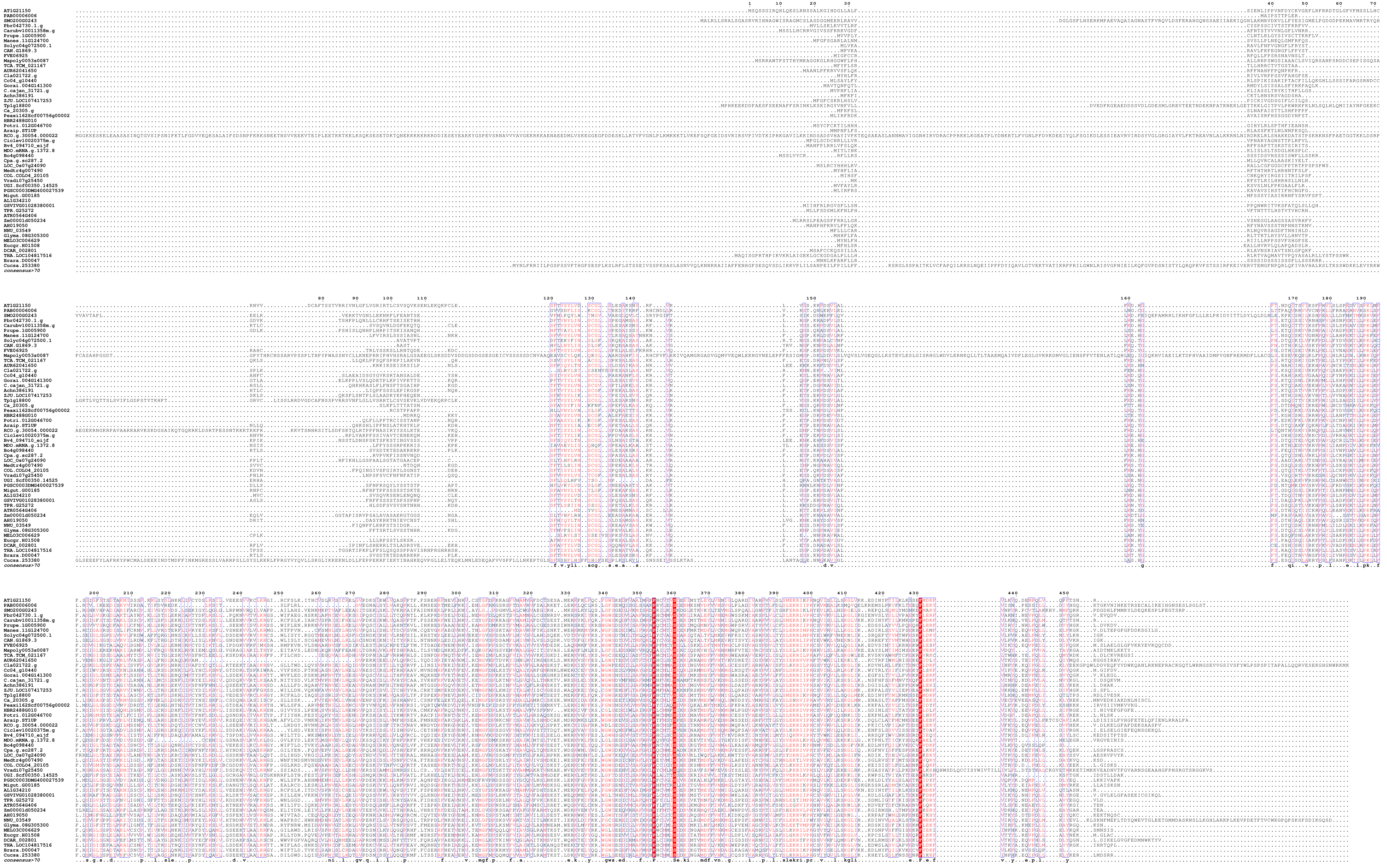

B

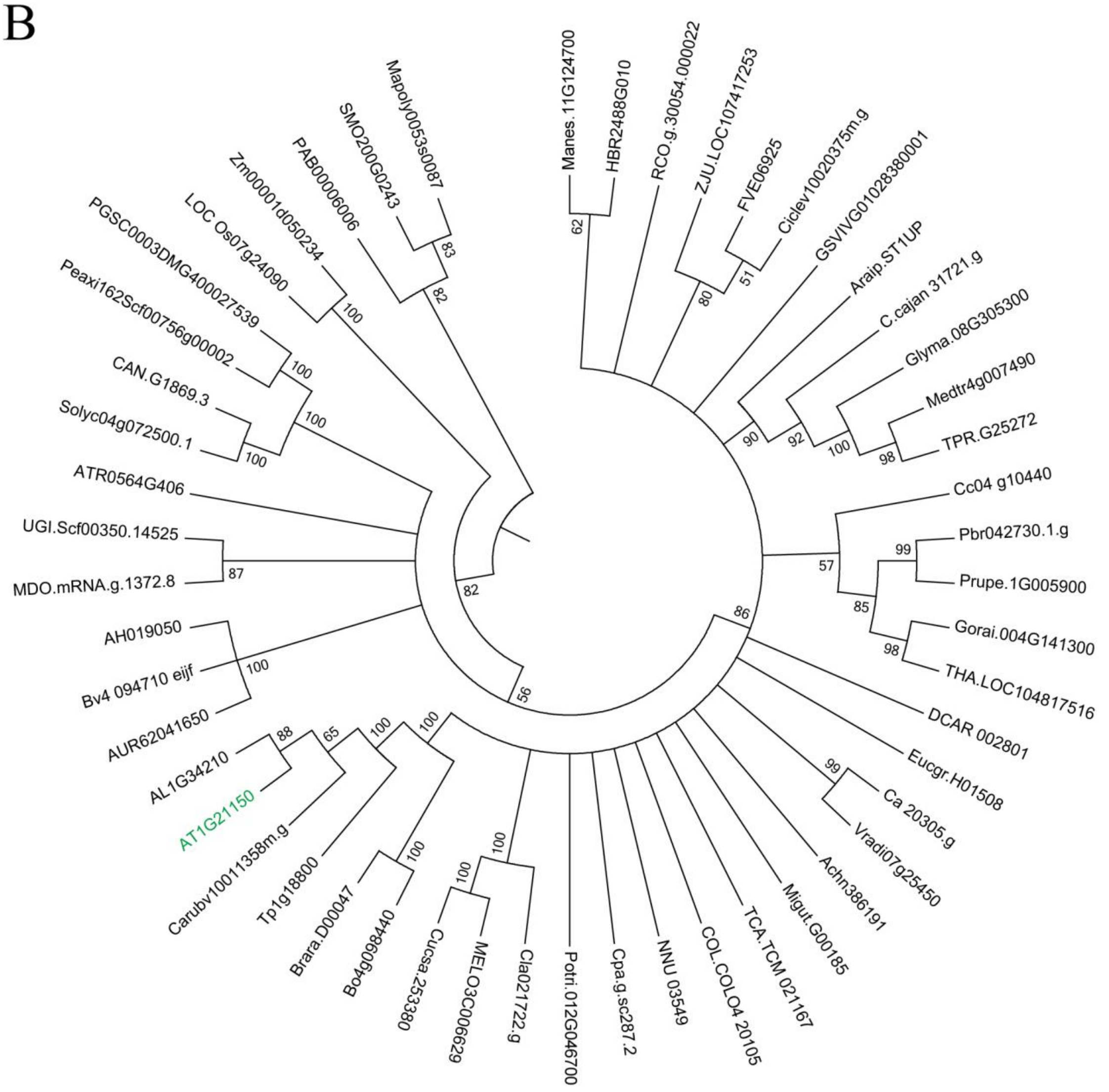

Fig. S1. Amino acid sequences alignment and Neighbor-joining phylogenetic tree of *mTERF27* ortholog genes in plants.

(A) Amino acid sequences alignment of *mTERF27* ortholog genes in plants.

(B) Neighbor-joining phylogenetic tree of Fig. S1A. Numbers on the nodes refer to bootstrap support values.

Species abbreviations are Achn, *Actinidia chinensis*; AH, Amaranthus hypochondriacus; ATR, *Amborella trichopoda*; AL, *Arabidopsis lyrata*; Araip, *Arachis ipaensis*; AT, *Arabidopsis thaliana*; Bv, *Beta vulgaris*; Bo, *Brassica oleracea*; Brara, *Brassica rapa*; C.cajan, *Cajanus cajan*; Carub, *Capsella rubella*; CAN, *Capsicum annuum*; Cpa, *Carica papaya*; AUR, *Chenopodium quinoa*; Ca, *Cicer arietinum*; Cla, *Citrullus lanatus*; Cicle, *Citrus clementina*; Cc, *Coffea canephora*; COL, *Corchorus olitorius*; MELO, *Cucumis melo*; Cucsa, *Cucumis sativus L.*; DCAR, *Daucus carota*; Migut, *Erythranthe guttata*; Eucgr, *Eucalyptus grandis*; FVE, *Fragaria vesca*; Glyma, *Glycine max*; Gorai, *Gossypium raimondii*; HBR, *Hevea brasiliensis*; MDO, *Malus domestica*; Manes, *Manihot esculenta*; Mapoly, *Marchantia polymorpha*; Medtr, *Medicago truncatula*; NNU, *Nelumbo nucifera*; Os,

Oryza sativa ssp. japonica; Peaxi, *Petunia axillaris*; PAB, *Picea abies*; Potri, *Populus trichocarpa*; Prupe, *Prunus persica*; Pbr, *Pyrus bretschneideri*; RCO, *Ricinus communis*; Tp, *Schrenkiella parvula*; SMO, *Selaginella moellendorffii*; Solyc, *Solanum lycopersicum*; PGSC, *Solanum tuberosum*; THA, *Tarenaya hassleriana*; TCA, *Theobroma cacao*; TPR, *Trifolium pratense*; UGI, *Utricularia gibba*; Vradi, *Vigna radiata var. radiata*; GSVIV, *Vitis vinifera*; Zm, *Zea mays*; and ZJU, *Ziziphus jujuba*. Integrative orthologs data of *mTERF27* was from PLAZA.

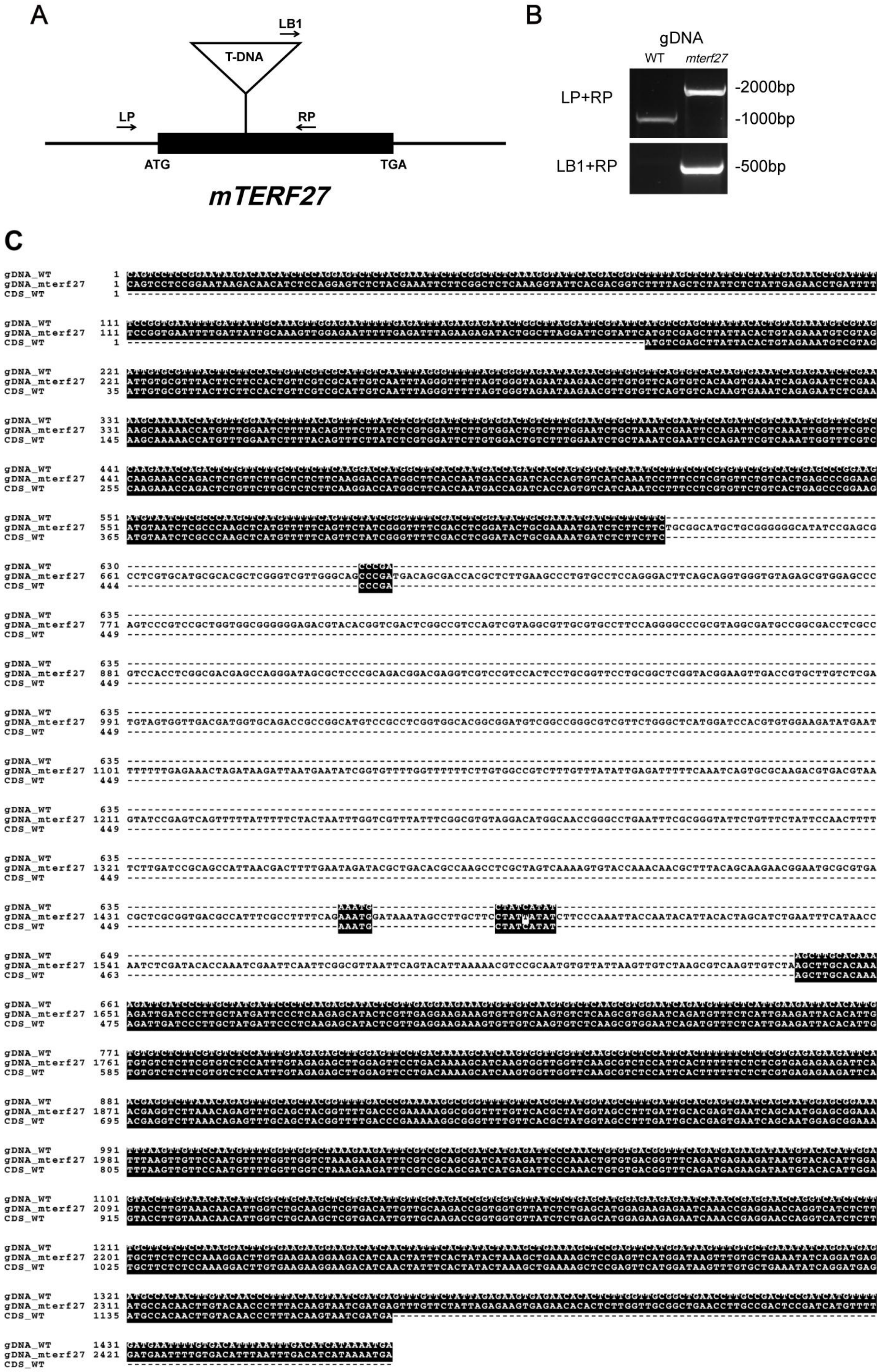
A
LB1
T-DNA
LP
RP
ATG
TGA
mTERF27
B
gDNA
WT
mterf27
LP+RP
-2000bp
-1000bp
LB1+RP
-500bp
C
gDNA_WT
gDNA_mterf27
CDS_WT

Fig. S2. Sequences the transfer DNA (T-DNA) insertion point in the *mterf27* mutant.

(A) Structure of *Arabidopsis* mTERF27 showing the T-DNA insertion site in the *mterf27* mutant. LP and RP indicate the forward and reverse PCR primers, respectively. LB1, plasmid primer.

(B) PCR amplifications confirming the presence of the T-DNA insertion site in the *mterf27* mutant. gDNA, genomic DNA.

(C) The T-DNA sequence in *mterf27*.

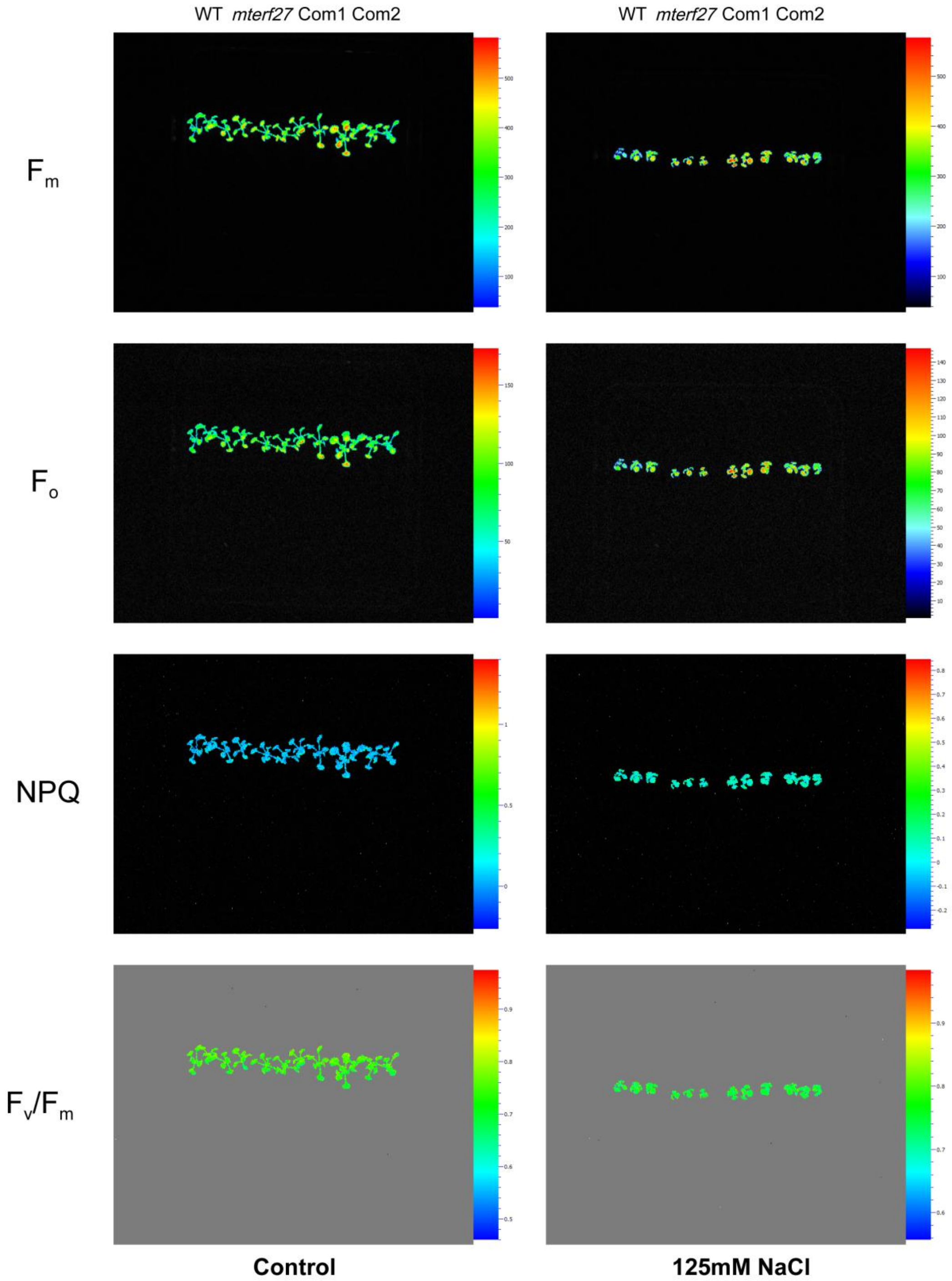

Fig. S3. Chlorophyll fluorescence analysis of the wild-type (WT) and *mterf27* plants shown in Fig. 2D.

mTERF27-complemented lines (Com1 and Com2). F_m, maximal fluorescence; F_o, minimal fluorescence; F_v/F_m, maximum efficiency of PSII photochemistry; NPQ, non-photochemical quenching.

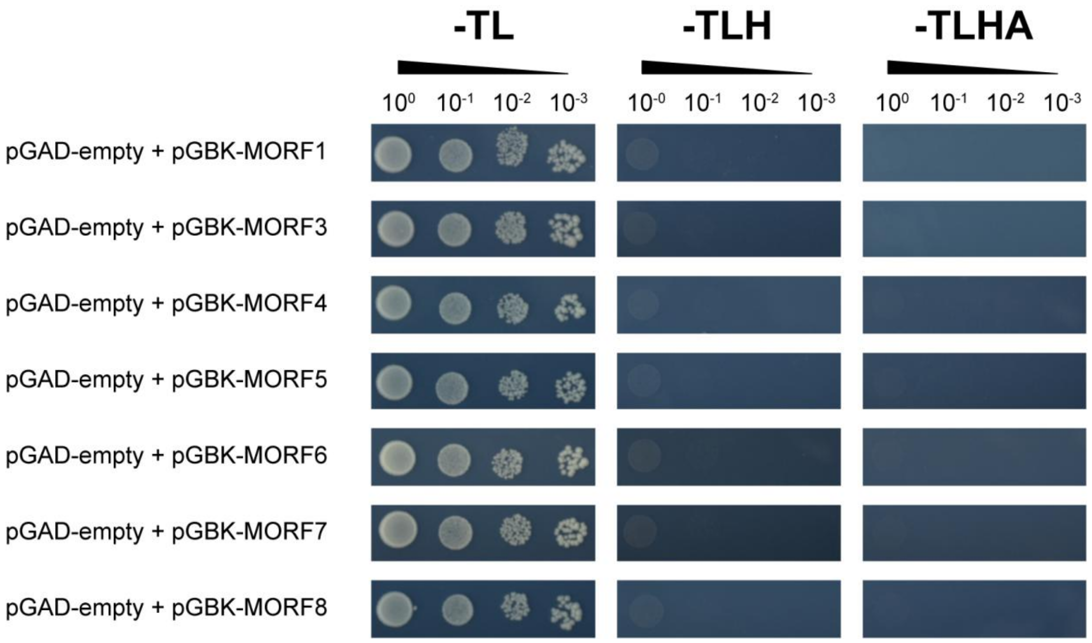

Fig. S4. Negative controls for the yeast two-hybrid assay used to determine protein interactions in Fig. 4A.

Table S1. Primers used in this study.

Names	Primers
mTERF27-F	ATGTCGAGCTTATTACACTGTAGAAATGTC
mTERF27-R	TCATCGATTACTTGTAAAGGGTTGTACAAGT
LP	CTCGTGTATGAGCCAGTCCTC
RP	ATTCACTCGTGCAATCAAAGG
LB1	GCCTTTTCAGAAATGGATAAATAGCCTTGCTTCC
mTERF27-BP-F	GGGGACAAGTTTGTACAAAAAAGCAGGCTCTATGTCGAGCTTATTACACTGTAGAA
mTERF27-BP-R	GGGGACCACTTTGTACAAGAAAGCTGGGTATCGATTACTTGTAAAGGGTTGTAC
mTERF27-qPCR-F	ACCATGGCTTCACCAATGACCAG
mTERF27-qPCR-R	TACATCTTCCGGGCTCAGTGAC
MORF1-BP-F	GGGGACAAGTTTGTACAAAAAAGCAGGCTCTATGGCTATGATATCTCACCGTCTC
MORF1-BP-R	GGGGACCACTTTGTACAAGAAAGCTGGGTAATGCCTGATGAGGATATCAAAAAAG
MORF3-BP-F	GGGGACAAGTTTGTACAAAAAAGCAGGCTCTATGGCTCTTATCAGCACACGG
MORF3-BP-R	GGGGACCACTTTGTACAAGAAAGCTGGGTAAGCACTGGGCTTGTTGAAC
MORF4-BP-F	GGGGACAAGTTTGTACAAAAAAGCAGGCTCTATGGCCATGTTCTCGCATCG
MORF4-BP-R	GGGGACCACTTTGTACAAGAAAGCTGGGTATATTCGAGAATTTGGTTTTCTGGTTTC
MORF5-BP-F	GGGGACAAGTTTGTACAAAAAAGCAGGCTCTATGGCGAAAACCCTAGCT
MORF5-BP-R	GGGGACCACTTTGTACAAGAAAGCTGGGTAACGCATGTTCTCTCTTCTTCG
MORF6-BP-F	GGGGACAAGTTTGTACAAAAAAGCAGGCTCTATGGCGAAAACTCTATCCCG
MORF6-BP-R	GGGGACCACTTTGTACAAGAAAGCTGGGTAACGCATGTTCTCCCTCCG
MORF8-BP-F	GGGGACAAGTTTGTACAAAAAAGCAGGCTCTATGGCGACGCATACCATTTCTC
MORF8-BP-R	GGGGACCACTTTGTACAAGAAAGCTGGGTAACCCTGGTAGGGGTTGCC
mTERF27-GFP-F	CGGGATCCATGTCGAGCTTATTACACTGTAGAAATGTC
mTERF27-GFP-R	CGGGATCCTCATCGATTACTTGTAAAGGGTTGTACAAGT

mTERF27-LUC-F	GCGTCGACATGTCGAGCTTATTACACTGTAGAAATGTC
mTERF27-LUC-R	GCGTCGACTCATCGATTACTTGTAAAGGGTTGTACAAGT
MORF8-LUC-F	GGGGTACCATGGCGACGCATACCATTTCTC
MORF8-LUC-R	GGACTAGTACCCTGGTAGGGGTTGCC
MEF13-LUC-F	GCGTCGACATGGCGGAGAGTCTCAGATTAT
MEF13-LUC-R	GCGTCGACGCAGTAACACCATTGGTCCTCT
MORF1-LUC-F	GGGGTACCATGGCTATGATATCTCACCGTCTC
MORF1-LUC-R	GGACTAGTATGCCTGATGAGGATATCAAAAAAG
